# Disordered eating risk and well-being in women with lipedema

**DOI:** 10.3389/fgwh.2026.1720708

**Published:** 2026-02-13

**Authors:** Monika Kunzová

**Affiliations:** 1 Department of Public Health, Faculty of Medicine, Masaryk University, Brno, Czechia; 2 International Clinical Research Center (ICRC), St Anne’s University Hospital (FNUSA) Brno, Brno, Czechia

**Keywords:** EAT-26, eating attitudes, lipedema, psychological well-being, WHO-5, women's health

## Abstract

**Background:**

Lipedema is a chronic adipose tissue disorder predominantly affecting women and is frequently misclassified as obesity. While its physical manifestations are increasingly recognized, less attention has been paid to eating attitudes and psychological well-being in this population. The objective of this study was to descriptively explore eating attitudes and psychological well-being in women with lipedema.

**Methods:**

This exploratory cross-sectional study used an anonymous online survey to describe eating attitudes and psychological well-being in women with lipedema. A total of 47 participants completed the Eating Attitudes Test (EAT-26) and the World Health Organization-5 Well-Being Index (WHO-5). Descriptive statistics were used to summarize screening indicators of disordered eating risk and reduced psychological well-being.

**Results:**

Approximately two-thirds of participants scored at or above the EAT-26 screening cut-off, reflecting elevated screening indicators of disordered eating risk. When behavioral risk indicators were included, over 70% screened positive according to EAT-26 criteria. Reduced psychological well-being (as indicated by a WHO-5 score of ≤50) was observed in about one-fifth of the sample.

**Conclusion:**

In this exploratory sample of women with lipedema, elevated screening indicators of disordered eating risk and reduced psychological well-being were commonly observed. These findings offer preliminary insights suggesting that eating-related risk and reduced well-being may be prevalent in this population. Further research using larger, clinically verified samples is needed to better understand the psychological aspects of lipedema.

## Introduction

Lipedema is a chronic adipose tissue disorder that predominantly affects women and is characterized by a symmetrical accumulation of subcutaneous fat, most commonly in the lower extremities, with relative sparing of the hands and feet. Typical clinical features include tenderness, easy bruising, and a sensation of heaviness (1, 2). Although its underlying pathophysiology remains incompletely understood, lipedema is increasingly recognized as a distinct clinical entity rather than a subtype of obesity (3, 4). Nevertheless, delayed diagnosis and misclassification as obesity remain common, often leading to repeated and unsuccessful weight-loss interventions (2, 5).

Beyond its physical manifestations, lipedema has been associated with psychosocial burden, including body image concerns, frustration related to weight management, and perceived stigmatization in healthcare settings (6–9). Such experiences may influence eating-related attitudes and behaviors; however, empirical data describing eating attitudes and psychological well-being in women with lipedema are still limited. Previous studies have primarily focused on quality of life and symptom burden, while eating-related outcomes have received comparatively less systematic attention (10–13).

Disordered eating behaviors, such as restrictive dieting or episodic loss of control over eating, have been described in populations experiencing chronic weight-related concerns or body image distress (14–16). In the context of lipedema, it remains unclear whether eating-related difficulties vary according to clinical characteristics of the condition, such as disease stage or type, or whether they appear relatively independent of physical symptom severity (17–19). A descriptive examination of eating attitudes alongside psychological well-being may therefore provide useful initial insight into the psychosocial profile of women with lipedema.

Therefore, the aim of this exploratory descriptive study was to characterize eating attitudes and psychological well-being in women with lipedema, and to descriptively examine whether these outcomes vary according to clinical characteristics of the condition.

## Methods

Between August and September 2025, a cross-sectional online survey was conducted among women in Czechia who either had a confirmed lipedema diagnosis or reported typical symptoms consistent with the condition. Given the absence of a national registry for lipedema, the sample size was determined pragmatically, based on recruitment feasibility rather than formal power estimation. Considering the exploratory character of this project, the main goal was to engage as many participants as possible within the data collection period.

Participants were recruited via a Czech Facebook community for individuals affected by lipedema, which comprised approximately 3,000 members at the time of recruitment. Participation was voluntary and uncompensated. Inclusion criteria were female sex and either a self-reported physician-confirmed diagnosis of lipedema or self-reported symptoms consistent with established diagnostic features.While social media–based recruitment may introduce self-selection bias, this approach remains a practical and widely accepted method for reaching individuals with rare or underdiagnosed conditions, as demonstrated in prior research (9).

### Assessment of lipedema characteristics

Participants reported whether their lipedema diagnosis had been confirmed by a physician. Those without physician confirmation completed a brief checklist assessing hallmark features of lipedema, including symmetrical fat accumulation in the extremities, tenderness or pain, easy bruising, heaviness of the legs, and sparing of the hands and feet, based on previously published descriptions (9). This checklist was used solely to describe the sample and was not employed for inferential analyses.

### Measures

#### Eating attitudes

Disordered eating risk was assessed using the Eating Attitudes Test (EAT-26) (20), a widely used screening instrument for eating disorder–related attitudes and behaviors. Total scores range from 0 to 78, with scores ≥20 indicating elevated risk of disordered eating. In addition to the total score, the behavioral items of the EAT-26 were used to identify the presence of eating-related risk behaviors, including binge eating, self-induced vomiting, laxative use, excessive exercise, or rapid weight loss within the previous six months.

The EAT-26 was used as a screening tool and not for diagnostic purposes.

#### Psychological well-being

Psychological well-being was assessed using the World Health Organization–5 Well-Being Index (WHO-5) (21), a validated measure of subjective psychological well-being over the preceding two weeks. Scores range from 0 to 100, with lower scores indicating poorer well-being. A score ≤50 was used to indicate reduced well-being, and a score ≤28 to indicate possible depressive symptomatology.

### Ethics

The study adhered to the principles of the Declaration of Helsinki and applicable data protection regulations (22, 23). Participation was anonymous, and no personally identifiable information was collected. All participants provided electronic informed consent prior to completing the questionnaire. According to institutional guidelines, formal ethical committee approval was not required for this anonymous, minimal-risk survey study.

### Statistical analysis

Statistical analyses were conducted using IBM SPSS Statistics, version 29.0. Descriptive statistics were used to summarize participant characteristics, eating attitudes, and psychological well-being. Continuous variables are presented as means and standard deviations, and categorical variables as frequencies and percentages. Internal consistency of the EAT-26 and WHO-5 was assessed using Cronbach's alpha. Internal consistency in the present sample was good for both instruments (WHO-5: Cronbach's *α* = 0.901; EAT-26: *α* = 0.862).

Exploratory comparisons of eating attitudes and well-being across selected clinical characteristics were performed descriptively. Given the exploratory nature of the study and the limited sample size, analyses were interpreted cautiously, and no adjustments for multiple comparisons were applied.

## Results

**Figure 1:**
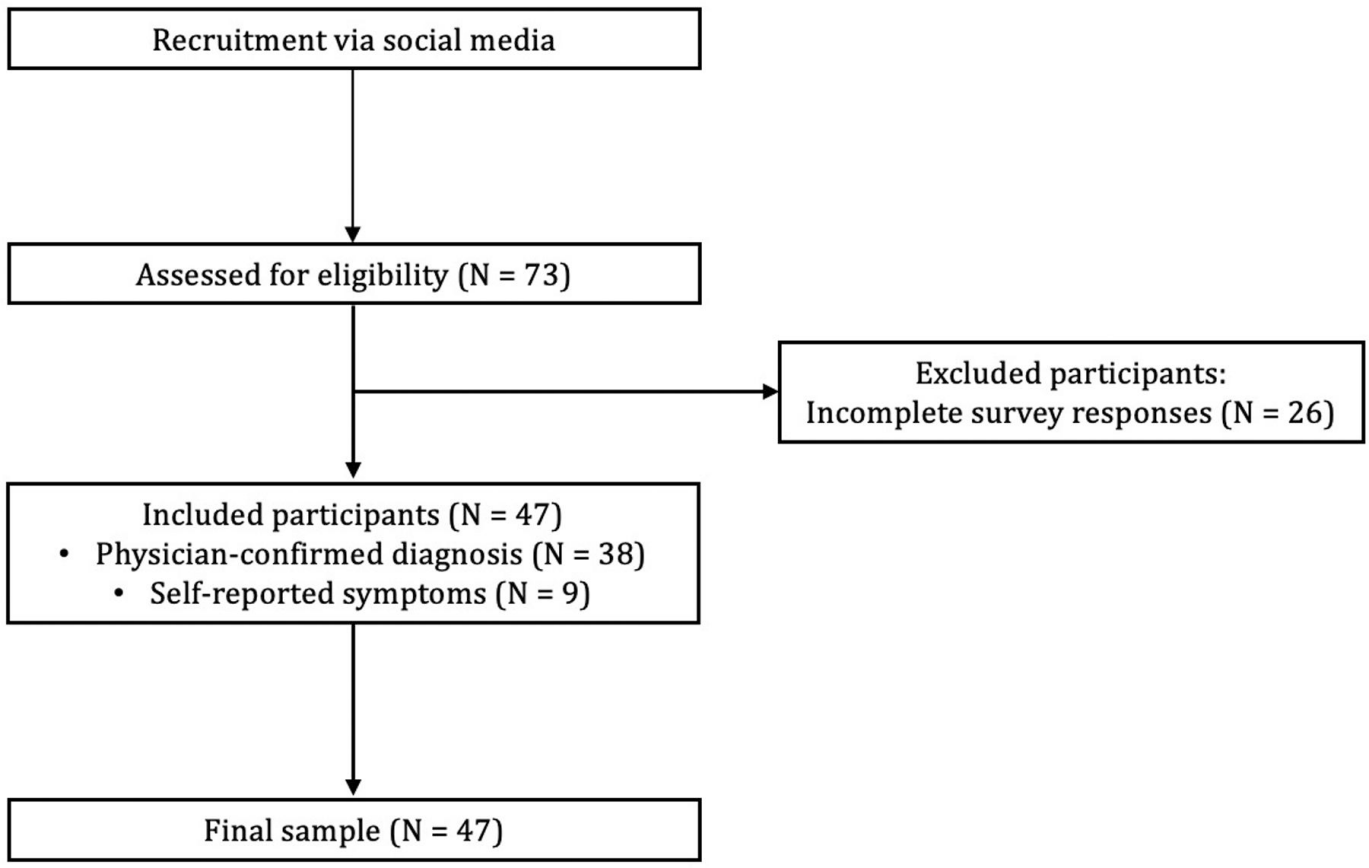
Workflow diagram of participant recruitment.

A total of 73 women initiated the online survey, of whom 47 completed all required sections and were included in the final analytic sample (Figure 1).

Participants ranged in age from 26 to 63 years (mean 41.5 ± 9.6 years). The majority of respondents (80.9%, *n* = 38) reported a physician-confirmed diagnosis of lipedema. Given the exploratory nature of the study and the similarity in self-reported characteristics between participants with and without physician confirmation, all respondents were included in the analyses.

Regarding disease-related characteristics, most participants reported symptom onset during puberty (59.6%), followed by pregnancy (12.8%) and menopause (10.6%), while 17.0% were unable to specify the onset period. Self-reported lipedema stage was distributed as follows: Stage I (29.8%), Stage II (53.2%), and Stage III (17.0%) (Table 1).

**Table 1:** Participants characteristics.

Variable	Mean ± SD/*n* (%)
Age (years)	41.49 ± 9.6
Confirmed diagnosis	38 (80.9)
Lipedema onset	
Puberty	28 (59.6)
Pregnancy	6 (12.8)
Menopause	5 (10.6)
Hard to tell	8 (17.0)
Lipedema stage	
Stage I	14 (29.8)
Stage II	25 (53.2)
Stage III	8 (17.0)
Education	
Upper secondary—vocational	5 (10.6)
Upper secondary—general/post-secondary	13 (27.7)
Tertiary	29 (61.7)

### Eating attitudes and disordered eating risk

**Figure 2:**
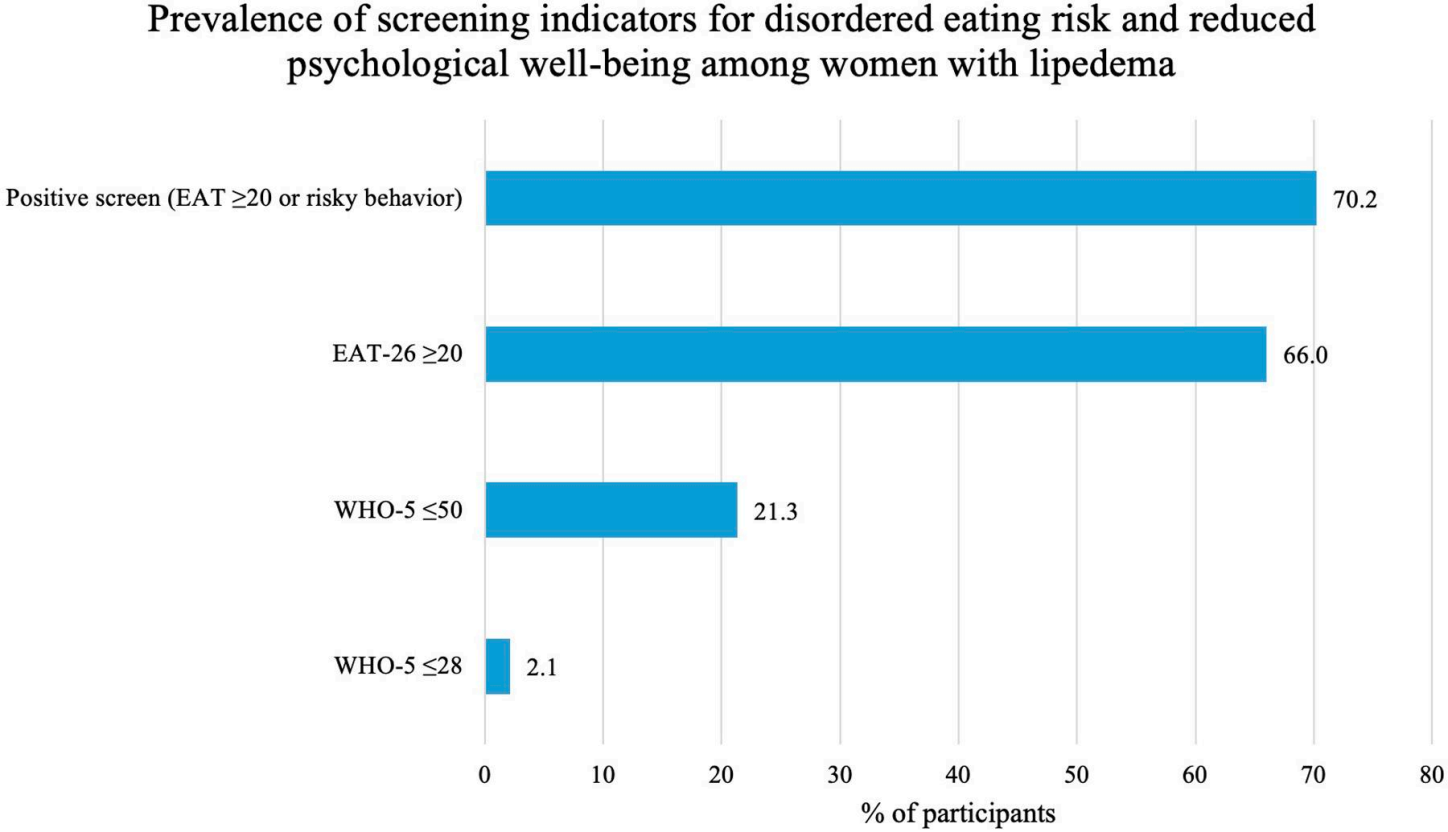
Prevalence of screening indicators for disordered eating risk and reduced psychological well-being among women with lipedema. Elevated disordered eating risk was defined as a positive screening result according to EAT-26 criteria (total score ≥20 or the presence of at least one eating-related risk behavior). Reduced psychological well-being was defined as a WHO-5 score ≤50, and possible depressive symptomatology as a WHO-5 score ≤28.

The mean EAT-26 total score in the sample was 26.7 ± 12.2. Overall, 66.0% of participants scored at or above the EAT-26 screening cut-off of 20, indicating elevated risk of disordered eating. When behavioral risk indicators were included, 70.2% of participants met criteria for a positive screening result (Table 2 and Figure 2).

**Table 2:** Eating attitudes test (EAT-26) scores and behavioral risk indicators among women with lipedema.

EAT-26 scores	Mean ± SD/*n* (%)
Total score (0–78)	26.7 ± 12.2
Score ≥20 (disordered eating risk)	31 (66.0)
Dieting subscale (0–39)	15.19 ± 7.1
Bulimia & Food preoccupation subscale (0–18)	4.38 ± 3.7
Oral control subscale (0–21)	3.34 ± 2.86
Behavioral Items (past 6 months)	
Binge eating ≥once per week	5 (10.6)
Self-induced vomiting ≥once per week	0 (0.0)
Use of laxatives, diet pills, or diuretics ≥once per week	2 (4.3)
Exercising >60 min daily to control weight	4 (8.5)
Weight loss ≥9 kg in past 6 months	9 (19.1)
Positive screen (EAT-26 ≥20 or ≥1 risky behavior)	33 (70.2)

Scores ≥20 on the EAT-26 indicate elevated risk of disordered eating. Behavioral items correspond to the EAT-26 Part C, assessing the frequency of compensatory or weight-control behaviors during the previous six months.

Among behavioral items, binge eating at least once per week was reported by 10.6% of participants, excessive exercise by 8.5%, and the use of weight-control agents by 4.3%. No participants reported self-induced vomiting.

To summarize the prevalence of elevated disordered eating risk and reduced psychological well-being in the sample, Figure 2 presents the proportion of participants screening positive on the EAT-26 and WHO-5 measures.

### Psychological well-being

The mean WHO-5 score was 46.9 ± 21.7. Reduced psychological well-being (WHO-5 ≤ 50) was observed in 21.3% of participants, while 2.1% scored at or below the threshold indicative of possible depressive symptoms (WHO-5 ≤ 28) (Table 3).

**Table 3:** Psychological well-being (wHO-5) scores and prevalence of reduced well-being in women with lipedema.

Variable	Mean ± SD/*n* (%)
Total score (0–100)	46.89 ± 21.7
≤50 (reduced well-being)	10 (21.3)
≤28 (indicative of depressive symptoms)	1 (2.1)

Scores range from 0 to 100, with higher values indicating greater well-being. A total score ≤50 suggests reduced psychological well-being, and ≤28 indicates a high risk of depressive symptoms.

### Eating attitudes and well-being across clinical characteristics

Exploratory descriptive comparisons did not indicate obvious differences in EAT-26 or WHO-5 scores across self-reported stages or types; these comparisons were not powered to detect group differences and should be interpreted cautiously.

## Discussion

In this exploratory descriptive study of women with lipedema, a high prevalence of elevated disordered eating risk was observed, with approximately two-thirds of participants scoring above the EAT-26 screening cut-off. In addition, reduced psychological well-being, as measured by the WHO-5, was identified in approximately one-fifth of the sample. These findings provide a descriptive overview of eating attitudes and psychological well-being in a group of women affected by lipedema.

The proportion of participants screening positive for disordered eating risk in this study appears higher than estimates typically reported in general female populations, where approximately 10%–20% score above the EAT-26 cut-off in community samples (24, 25) Previous studies in lipedema have similarly reported elevated levels of weight-related distress and problematic eating attitudes (11, 12, 17, 26) Lipedema is linked to significant psychosocial burdens, including reduced emotional functioning and quality of life, even when compared to other chronic conditions (27–29). However, given the exploratory design and modest sample size, direct comparisons with population-based studies should be interpreted with caution.

Reduced psychological well-being was observed in a subset of participants, while most women reported WHO-5 scores above the threshold indicative of possible depressive symptomatology. This pattern aligns with prior research suggesting that psychosocial burden is common in lipedema, although its magnitude and clinical relevance vary across individuals (9, 17, 18). Psychological distress in lipedema may be influenced not only by physical symptoms but also by psychosocial factors such as weight stigma, internalized bias, and repeated experiences of unsuccessful weight management (19). The present findings contribute to this literature by providing descriptive data on well-being using a standardized screening instrument.

In addition to questionnaire-based studies, rare clinical case reports have described diagnosed eating disorders in women with lipedema, further illustrating the complex relationship between adipose tissue disorders and eating-related psychopathology. For example, individual cases of anorexia nervosa have been reported in women with lipedema, in which restrictive eating behaviors coexisted with persistent disproportionate fat accumulation resistant to weight loss despite severe caloric restriction (11, 30). While these reports are not generalizable, they indicate that eating-related issues in lipedema may not follow typical weight patterns and can arise despite ongoing fat accumulation. This highlights the need for increased awareness of eating difficulties in this population, even without formal eating disorder diagnoses.

Importantly, this study was not designed to test causal relationships or underlying mechanisms. Rather, it offers an initial descriptive characterization of eating attitudes and psychological well-being in women with lipedema. The absence of inferential conclusions underscores the need for further research using larger, clinically verified samples and longitudinal designs to clarify the relationships between physical characteristics of lipedema, eating-related behaviors, and psychological outcomes.

Several methodological limitations should be noted when interpreting these findings. The modest sample size, recruited via online platforms, may limit generalizability and introduce self-selection bias. The higher educational attainment in the sample suggests that participants may represent women with greater health awareness, which could influence their eating-related attitudes and psychological well-being.

Additionally, about one-third of individuals who started the survey did not complete it, which may reflect the sensitive nature of questions regarding eating behaviors and mental health, leading to underrepresentation of those experiencing significant distress. All data were self-reported, including lipedema diagnosis and psychological outcomes, which could introduce recall or reporting bias. The EAT-26 and WHO-5 were chosen as brief, well-established self-report screening tools due to their strong psychometric support in research. Although these instruments are reliable and commonly used for evaluating eating attitudes and well-being in various populations, they are not explicitly designed for lipedema. Their dependence on self-reported data and the absence of constructs tailored to lipedema may limit their ability to identify unique factors contributing to eating-related distress in this population. The cross-sectional design of the study also prevents causal inference, limiting interpretation to indicators of risk rather than confirmed diagnoses.

Despite these limitations, this study offers new insights suggesting that elevated eating-related risk and reduced psychological well-being may be common among women with lipedema, supporting the need for larger, clinically verified and longitudinal research.

## Conclusions

In this exploratory descriptive study, elevated disordered eating risk and reduced psychological well-being were frequently observed among women with lipedema. Given the exploratory design and modest sample size, these results should be interpreted cautiously as screening indicators rather than clinical diagnoses. Overall, the findings offer new insights suggesting that eating-related risk and reduced well-being may be common in this population. Further research using larger, clinically verified and longitudinal samples is needed to better understand the psychological aspects of lipedema and their relevance for patient-centered care.

## Data Availability

The raw data supporting the conclusions of this article will be made available by the authors, without undue reservation.
